# Texture synthesis of ecological plant protection image based on convolution neural network

**DOI:** 10.3389/fpls.2022.1035077

**Published:** 2022-10-18

**Authors:** Libing Hu, Fei Zhou, Xianjun Fu

**Affiliations:** ^1^ Book Information Center, Zhejiang College of Security Technology, Wenzhou, China; ^2^ Business School, Wenzhou University, Wenzhou, China; ^3^ College of Artificial Intelligence, Zhejiang College of Security Technology, Wenzhou, China

**Keywords:** convolutional neural network, ecological plant protection, image processing, texture synthesis method, realistic rendering technology

## Abstract

Texture synthesis technology is an important realistic rendering technology. Texture synthesis technology also has a good application prospect in image rendering and other fields. Convolutional neural network is a very popular technology in recent years. Convolutional neural network model can learn the features in data and realize intelligent processing through the feature learning in data. Later, with the rapid improvement of convolutional neural network, texture synthesis technology based on neural network came into being. The purpose of this paper is to study the texture synthesis method of ecological plant protection image based on convolutional neural network. By studying the context and research implications, the definition of textures as well as texture synthesis methods, convolutional neural networks, and based on convolutional neural network. In the experiment, the experimental environment is established, and the subjective evaluation and objective evaluation of the image texture synthesis method experiment are investigated and studied by using swap algorithm. The experimental results show that the method used in this paper is superior to other methods.

## Introduction

Convolutional neural network is proposed according to the visual cognitive mechanism. Due to its local connection and weight sharing characteristics and the local translation invariance characteristics brought by the idea of spatial downsampling, it has made outstanding achievements in computer vision, speech recognition and other fields in recent years. The application of convolutional neural network to realize texture synthesis has also become one of the current research hotspots (Watanabe et al., 2018). Images in nature or human real life contain various textures, and these textures have different characteristics. A part of these texture images is intercepted, whether it is a regular texture image or an irregular texture image, a new texture image can be formed.

As a significant branch of computer graphics, texture synthesis has always been the focus of researchers. The purpose of the Medeiros f a study was to determine whether the retinal nerve fiber layer thickness prediction obtained by the deep learning model applied to the fundus photos could detect progressive glaucoma changes over time. Design a retrospective cohort study of participants on color fundus photographs and spectral domain optical coherence tomography. Methods a deep learning convolutional neural network was trained to evaluate the fundus photos and predict the global retinal nerve fiber layer thickness measurement by tomography. The model was then tested on an independent eye sample with longitudinal follow-up by fundus photography and tomography. The ability to detect eyes with statistically significant tomographic change slopes was assessed by the subject operating characteristic curve. The repeatability of retinal nerve fiber layer thickness prediction was studied by measuring results obtained from multiple photos taken on the same day (Medeiros et al., 2021). Large scenes such as the building facade and other building structures of Labrie Larrivee F usually contain repetitive elements, such as the same window and brick patterns. A new method is proposed to improve the resolution and geometry of 3D meshes of large scenes with such repetitive elements. By using the structure from motion reconstruction and the ready-made depth sensor, the method captures small samples of the scene with high resolution and automatically extends the information to similar areas of the scene. The method uses RGB and SFM depth information as guidance, simple geometry as canvas, and powerful image-based texture synthesis method to expand the high-resolution mesh. The final result improves the standard SFM reconstruction with higher detail. Compared with full RGBD reconstruction, the method benefits from reduced manual labor and can be much cheaper than lidar based solutions (Labrie-Larrivee et al., 2019). The research of texture synthesis technology has made great progress, and a large number of new methods have appeared which lays a more solid theoretical foundation for digital image restoration.

In the second chapter, according to the background and research significance of the topic, the definition of texture and texture synthesis method, convolutional neural network, and the texture synthesis method of ecological plant protection image based on convolutional neural network are described. In the experiment of the third chapter, the experimental environment is established, and the subjective evaluation and objective evaluation of the image texture synthesis method experiment are investigated and studied in the fourth chapter by using the Swap algorithm. The experimental results show that the method used in this paper outperforms other methods.

## Research on texture synthesis of ecological plant protection image based on convolution neural network

### Topic background and research significance

The issue of image realism has always been the main course in computer graphics, and is also the focus of scientific researchers (Sardar and Tileubaeva, 2019; Larras et al., 2022). At first, people directly expressed the subtle structure of objective things through geometric models. However, the objective world is complex and changeable. This method has a large amount of calculation and modeling is also very difficult to meet the actual needs. In order to make up for the deficiency of geometric model expression, people combine the image processing technology with computer graphics technology, and use the image processing, analysis and synthesis technology to realize the realistic display of images, and have made a long-term improvement.

In recent years, texture synthesis technology, as an important image-based realistic rendering technology, has always received the attention of researchers and has high practical application value (Jassim and Harte, 2020; Shenson et al., 2021). Deep learning refers to interpreting data by simulating the thinking mode of human brain through different machine learning algorithms. It is a learning algorithm closest to human brain and has become one of the important branches of artificial intelligence research. Unlike traditional neural networks, deep learning networks are deeper in network level, larger in scale and higher in complexity. They can count large-scale data, learn fundamental characteristics from massive data, achieve breakthroughs in the field of artificial intelligence, and lead an innovative revolutionary upsurge. Convolutional neural network has the characteristics of weight sharing and pooling layer dimension reduction, which greatly shortens the training time of network model and improves the training time of model. Therefore, many remarkable research achievements have been made in image recognition and image processing.

### Definition of texture and texture synthesis method

Texture is a concept often used in the field of computer graphics and photorealistic rendering. At present, it is generally defined as: the expression on the surface of any object is regarded as texture (Hsu and Yeh, 2018; Nikitina et al., 2018). In the field of computer graphics and image processing, people agree with the definition that texture refers to repeating the basic texture element - texel.

The basic constituent unit of texel is pixel, and texture refers to the type of image containing a special attribute, which is the realization of a random process, which is local and stable (Williams et al., 2020; Preston et al., 2022). Texture image is different from ordinary image because of its locality and stability. Because of the complex changes of everything in the world, the classification of texture is also changeable. Texture can be obtained by image scanning, manual drawing, and computer image generation technology. According to the classification of texture synthesis, texture synthesis technology can be divided into three types: texture mapping method, process texture synthesis method and sample based texture synthesis method (Rsa et al., 2020; Borovik et al., 2022).

### Convolutional neural network

Convolutional neural network is the most typical type of neural network, which is generally composed of input layer, hidden layer and output layer (Rafizah et al., 2018; Gribanov et al., 2021). The parameters of convolution layer include the number of input channels, the number of output channels, the size of convolution core, step size, filling, etc. among the many parameters of convolution layer, three are more important, which determine the function of convolution layer to a certain extent, namely, the number of convolution cores, the number of input channels and the number of output channels. Generally speaking, the convolution layer is composed of multiple convolution kernels. Any element in the convolution kernel is a numerical type weight coefficient that can be changed. In most cases, each convolution kernel corresponds to a numerical type weight coefficient, which is called the deviation amount. The coefficients of each convolution kernel in the convolution layer are weighted sum of multiple weight coefficients in the previous layer. In this sense, the convolution layer is a special feedforward neural network unit. Specifically, when the convolution kernel size is 1, the step size is 1, and no filling is performed, the convolution operation is equivalent to matrix multiplication, and the convolution layer is equivalent to a fully connected network. Some convolution networks use some special convolution structures, such as transposed convolution, extended convolution and separable convolution. These convolutions have their own characteristics and advantages, but also have some shortcomings. In most network models dealing with various tasks, there are often various convolution structures and basic units. Through mutual cooperation, a complex large-scale network that can complete the target task is constructed.

### Texture synthesis method of ecological plant protection image based on convolutional neural network

The early texture synthesis technology based on neural network is mainly based on the theory of artificial neural network. A texture synthesis method based on continuous Hopfield network is proposed, which connects the neurons with the pixels of the image to synthesize different two tone and gray tone texture images; The theory of BP neural network is applied to the ecological plant protection texture image generation, and the application of artificial neural network in the ecological plant protection texture image synthesis is studied by using the analysis and feature extraction of the ecological plant protection texture. However, the effect of texture generation based on simple artificial neural network is not very ideal, and the extraction of image features is still based on artificial analysis, which makes the design cycle relatively long (Stepanova et al., 2018; Esquivel-Castro et al., 2019).

In recent years, with the rise and rapid improvement of deep learning, texture synthesis based on convolutional neural network has also achieved a lot of research results. Based on VGg network, the loss function is added to the statistical feature distribution of the image. These statistical distributions are calculated by the Clem matrix. This method has been extended by many researchers. Combined with Markov random field, Gatys et al., the method is extended to the fusion of ecological plant protection images, and the synthetic effect of ecological plant protection images is more stable. A confrontation generation network with 5-layer full convolution neural network as generator and discriminator is constructed. The synthetic texture ecological plant protection image is more realistic. Although the network training time is long, the test time is only 0.2S.

## Investigation and research on texture synthesis of ecological plant protection image based on convolution neural network

### Experimental environment

The experimental environment in this paper is a general experimental environment for deep learning, in which GPU processor of company a and CPU processor of company B are used. The training of convolutional neural network model requires that the neural network model be loaded into the GPU processor and then trained. At the same time, set the batch size during training. Batch size is the number of data samples selected during training the neural network model.

### Research content

In the experiment, the subjective evaluation of the image texture synthesis method and the experiment will be investigated and analyzed, and finally the experimental results will be obtained.

### Swap algorithm

The swap algorithm operates directly on the feature maps extracted by the convolutional neural network to find the similarity between the feature maps extracted by the loss network. The input of the loss network includes the output of the texture network, that is *I*_1_ . The corresponding input model is *M*_1_ set in the previous text, which can be assumed as *M*_1_(*I*_1_) . The value in *M*_1_(*I*_1_) is composed of the characteristic diagram of each layer in *M*_1_ neural network, namely:


(1) $$M_{1}(I_{1})=\{F_{1}^{l},F_{2}^{l},F_{3}^{l},\ldots ,F_{n}^{l}\}(n-1,2,\ldots ,n)$$

Among them, $F_{n}^{l}$ represents the feature map of a layer in the *M*_1_ model; at the same time, the *I*_1_ corresponding to *I*_gt_ is used as an input and input to the neural network model red *M*_2_ , that is, *M*_2_(*I*_gt_) . The values in *M*_2_(*I*_gt_) consist of feature maps for each layer in the *M*_2_ model, namely:


(2) $$M_{2}(I_{\text{gt}})=\{f_{1}^{l},f_{2}^{l},f_{3}^{l},\ldots ,f_{n}^{l}\}(n-1,2,\ldots ,n)$$

Among them, $f_{n}^{l}$ represents the feature map of a certain layer in the model *M*_2_ .

Finally, the swap algorithm designed in this paper is used to perform the matching calculation. Here, swap can be viewed as a network layer similar to the convolutional layer. The formula is expressed as follows:


(3) $${\text{swap}}_{texture}=swap(M_{2}(I_{\text{gt}}),M_{1}(I_{1}))$$

## Analysis and research on texture synthesis of ecological plant protection image based on convolution neural network

### Subjective evaluation of image texture synthesis method experiment

The image (a) column is the original sample texture, and the image (b) column is the texture image generated by the method in this paper, the image (c) column is the texture image generated by the method of Gayts et al. By making a questionnaire, we select some students and teachers to participate in the evaluation of the generated texture image results, and we subjectively get the superiority of a certain method through their scores.

**Figure 1:**
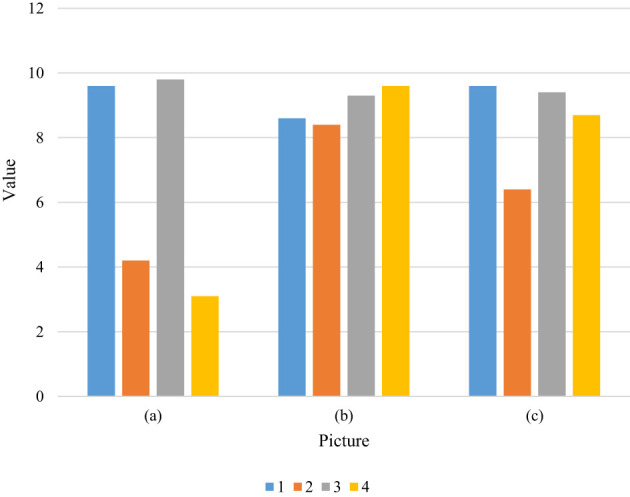
Evaluation data comparison is shown. The image (a) column is the original sample texture, the image (b) column is the texture image generated by this method, and the image (c) column is the text image generated by Gayts and other methods.

**Figure 2:**
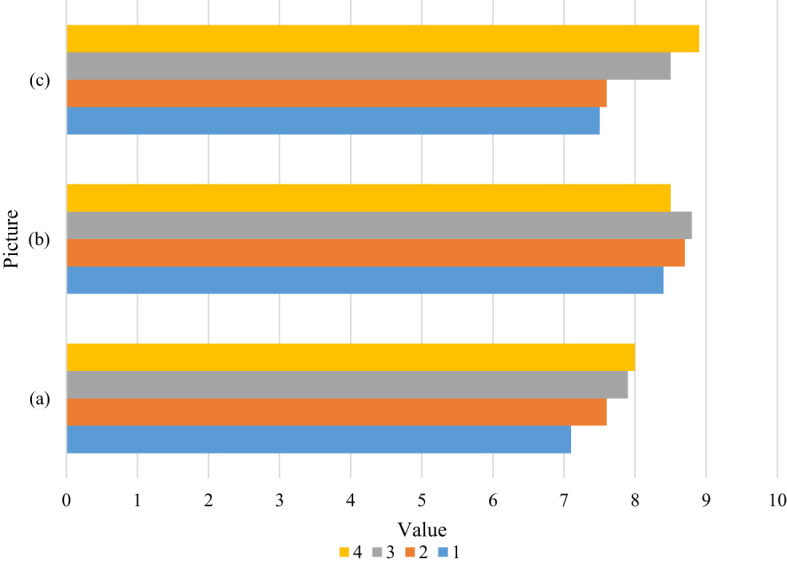
Subjective evaluation data for comparison. Using the subjective evaluation method, we obtained 2 evaluation sheets by randomly selecting 2 people. The 2 evaluation sheets have each person’s evaluation and score for the texture results. Through calculation, we can get: The average score of the method in this paper is 8.2 points. The method in image **(c)** has a highest score of 9.5.Although some texture synthesis results of other methods may be superior to our results, our method outperforms other methods in many texture synthesis image results.

Since the field of study and study of each teacher and student is different, this method can be regarded as an auxiliary evaluation method and cannot play a decisive role. Let these images be evaluated by 2 randomly selected people. The evaluation tables of these 2 people are shown in **Table 1** and **Figure 1**, and **Table 2** and **Figure 2**:

**Table 1:** Evaluation Table.

Picture	1	2	3	4
(a)	9.6	4.2	9.8	3.1
(b)	8.6	8.4	9.3	9.6
(c)	9.6	6.4	9.4	8.7

**Table 2:** Teacher evaluation form.

Picture	1	2	3	4
(a)	7.1	7.6	7.9	8.0
(b)	8.4	8.7	8.8	8.5
(c)	7.5	7.6	8.5	8.9

Therefore, we can conclude that our method has certain advantages compared with other methods. However, the use of subjective evaluation methods to evaluate the generated results has certain limitations, because the subjective evaluation due to the existence of human interference factors, due to personal experience, personal preferences, and differences in the knowledge level of each person will make the same texture synthesis result. There is a large difference in the evaluation of the subjective evaluation method, which will make the subjective evaluation method have a certain error and will not be very accurate. Therefore, an objective evaluation method is also introduced in this paper to ensure the objectivity of the generation effect evaluation results.

### Objective evaluation of image texture synthesis method experiment

**Figure 3:**
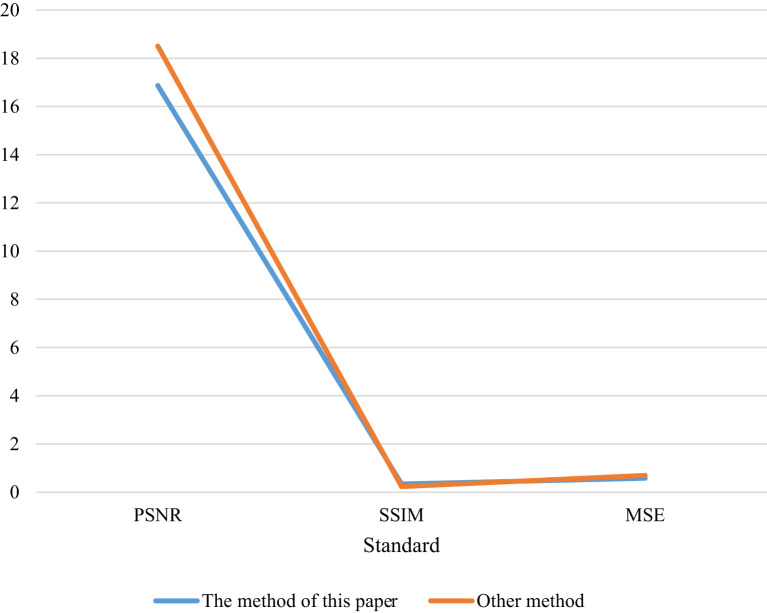
Image evaluation data in Figure.

**Figure 4:**
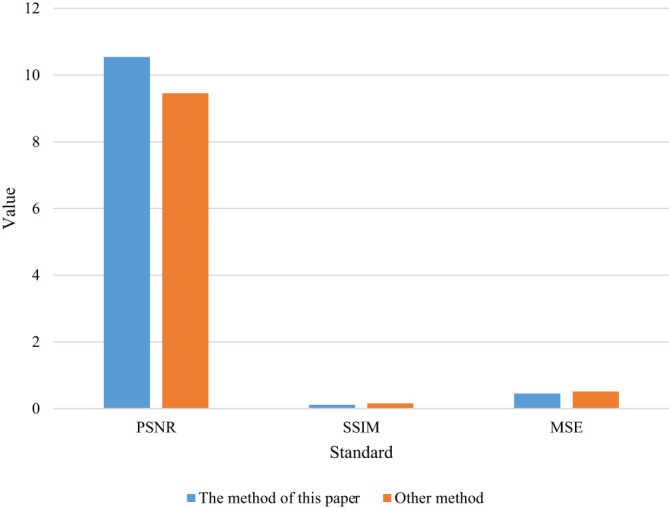
Objective evaluation of the data in Figure.

**Figure 5:**
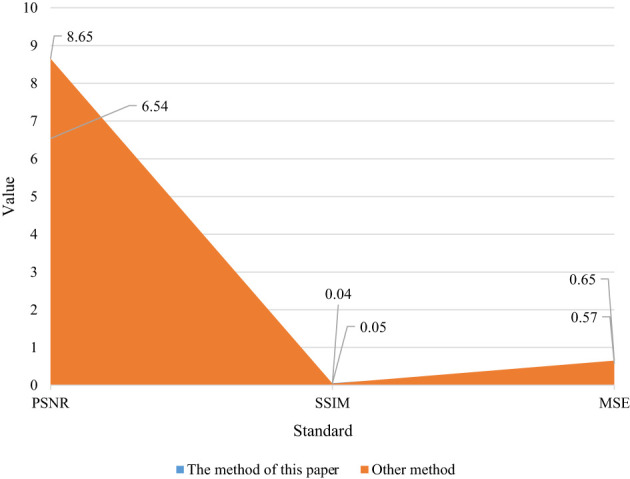
Comparison diagram of the data in row C. Through the evaluation of objective methods, it can be seen that the method in this paper can be close to other methods in some texture synthesis. The biggest innovation of our method is the synthesis at multiple scales, which surpasses other methods in this respect. The method in this paper is compared with other texture synthesis algorithms based on convolutional neural network, and the conclusions are drawn from the perspectives of subjective evaluation and objective evaluation. Through the evaluation of objective methods, it can be seen that the method in this paper can be close to other methods in some texture synthesis.

In this section, the objective evaluation methods used are introduced, these methods are: Mean Squared Error (MSE), Peak Signal-Noise Ration (PSNR), Structural Similarity (Structural Similarity, SSIM). Similarly, in the objective evaluation section, use the objective evaluation criteria to compare the parameters of the image evaluation table as shown in **Table 3** and **Figure 3**, and **Table 4** and **Figure 4**, **Table 5** and **Figure 5**:

**Table 3:** Image evaluation table.

Evaluation criterion	The method of this paper	Other method
PSNR	16.87	18.51
SSIM	0.34	0.23
MSE	0.57	0.69

**Table 4:** Objective evaluation table.

Evaluation criterion	The method of this paper	Other method
PSNR	10.54	9.45
SSIM	0.11	0.15
MSE	0.45	0.51

**Table 5:** Line C image evaluation table.

Evaluation criterion	The method of this paper	Other method
PSNR	6.54	8.65
SSIM	0.05	0.04
MSE	0.57	0.65

## Conclusions

The convolutional neural network model has made significant breakthroughs and achievements in many aspects, such as image synthesis, image classification, image segmentation and other fields. In this paper, the related things about texture synthesis algorithm are mainly introduced. Texture synthesis algorithms are divided into traditional and modern. There are some defects in the traditional texture synthesis method. For example, the texture synthesized in texture mapping may have seams that change; the problem in the procedural texture synthesis method is that new parameters need to be adjusted when a new texture is not generated., Different textures correspond to different parameters, which is a troublesome way to generate textures; the textures existing in the texture synthesis method based on sample images are the sample images needed to synthesize textures. If the selection is not appropriate, the synthesized textures may be The real feeling will be lost.

## Data availability statement

The original contributions presented in the study are included in the article/supplementary material. Further inquiries can be directed to the corresponding author.

## Author contributions

FZ: Conceptualization, Funding Acquisition, Resources, Supervision, Writing - Review and Editing. LH: Conceptualization, Methodology, Software, Investigation, Formal Analysis, Writing - Original Draft; XF: Visualization, Investigation; Resources, Supervision; Software, Validation. All authors contributed to the article and approved the submitted version.

## Conflict of interest

The authors declare that the research was conducted in the absence of any commercial or financial relationships that could be construed as a potential conflict of interest.

## Publisher’s note

All claims expressed in this article are solely those of the authors and do not necessarily represent those of their affiliated organizations, or those of the publisher, the editors and the reviewers. Any product that may be evaluated in this article, or claim that may be made by its manufacturer, is not guaranteed or endorsed by the publisher.

## References

[B1] Borovik P. Oestreicher V. Angelomé P. C. Barja B. C. Jobbágy M.. (2022). Room temperature synthesis of lanthanum phosphates with controlled nanotexture as host for Ln(III) through the epoxide route. J. Sol-Gel Sci. Technol. 102 (1), 279–287. doi: 10.1007/s10971-022-05744-w

[B2] Esquivel-Castro T. A. Martínez-Luévanos A. García-Cerda L. A. Contreras-Esquivel JC Pérez P. B. González Aguilera E. N.. (2019). Effect of the drying on morphology and texture of aerogels and zirconia cryogels. MRS Adv. 4 (64), 1–9. doi: 10.1557/adv.2019.450

[B3] Gribanov E. N. Gorshkov A. I. Sinitsyn E. A. Yu Khripunov V. Oskotskaya E. R. (2021). On the synthesis and morphology and formation peculiarities of an alumosilicate film on a substrate. J. Surf Invest. X-ray Synchrotron Neutron Tech 15 (1), 16–23. doi: 10.1134/S1027451021010079

[B4] Hsu P. K. Yeh J. (2018). PS02.070: Deep neural network to predict poor prognostic factors in patients with esophageal cancer. Dis. Esophagus 31 (13), 140–140. doi: 10.1093/dote/doy089.PS02.070

[B5] Jassim W. A. Harte N. (2020). Estimation of *a priori* signal-to-noise ratio using neurograms for speech enhancement. J. Acoust Soc. America 147 (6), 3830–3848. doi: 10.1121/10.0001324 32611151

[B6] Labrie-Larrivee F. Laurendeau D. Lalonde J. F. (2019). Depth texture synthesis for high-resolution reconstruction of large scenes. Mach. Vision Appl. 30 (4), 795–806. doi: 10.1007/s00138-019-01030-y

[B7] Larras F. Charles S. Chaumot A. Pelosi C. LeGall M. Mamy L.. (2022). A critical review of effect modeling for ecological risk assessment of plant protection products. Environ. Sci. Pollut. Res. 29 (29), 43448–43500. doi: 10.1007/s11356-022-19111-3 35391640

[B8] Medeiros F. A. Jammal A. A. Mariottoni E. B. (2021). Detection of progressive glaucomatous optic nerve damage on fundus photographs with deep learning. Ophthalmology 128 (3), 383–392. doi: 10.1016/j.ophtha.2020.07.045 32735906 PMC7386268

[B9] Nikitina M. A. Pchelkina V. A. Kuznetsova O. A. (2018). Technological solutions for intelligent data processing in the food industry[J]. Proc. Voronezh State Univ. Eng. Technol. 80 (2), 256–263. doi: 10.20914/2310-1202-2018-2-256-263

[B10] Preston F. G. Meng Y. Burgess J. Ferdousi M. Azmi S. Petropoulos I. N.. (2022). Artificial intelligence utilising corneal confocal microscopy for the diagnosis of peripheral neuropathy in diabetes mellitus and prediabetes. Diabetologia 65 (3), 457–466. doi: 10.1007/s00125-021-05617-x 34806115 PMC8803718

[B11] Rafizah Z. Omar N. A. Ibrahim N. (2018). Morphology and chemical structure of sn(Oct)2 thin layer added binder *via* sol gel method. Malaysian J. Anal Sci. 22 (2), 311–317. doi: 10.17576/mjas-2018-2202-17

[B12] Suarez A. R. Muñoz F. F. Bonelli P Cukierman A. L Larrondo S. A.. (2020). Hierarchical, template-free self-assembly morphologies in CeO2 synthesized *via* urea-hydrothermal method - ScienceDirect. Ceram Int. 46 (8), 11776–11785. doi: 10.1016/j.ceramint.2020.01.212

[B13] Sardar A. A. Tileubaeva Z. S. (2019). Influence of tillage methods and plant protection agents on the ecological parameters of soil cover and barley yield. Agric. Machinery Technol. 13 (3), 8–10. doi: 10.22314/2073-7599-2019-13-3-8-10

[B14] Shenson J. A. Liu G. S. Farrell J. Blevins N. H. (2021). Multispectral imaging for automated tissue identification of normal human surgical Specimens:[J]. Otolaryngol–Head Neck Surg. 164 (2), 328–335. doi: 10.1177/0194599820941013 32838646

[B15] Stepanova L. N. Belskaya O. B. Vasilevich A. V. Leont’eva N. N. Baklanova O. N. Likholobov V. A.. (2018). Effect of the composition of initial components and the conditions of activation on the mechanochemical synthesis of magnesium–aluminum layered double hydroxides. Kinet Catal 59 (4), 521–531. doi: 10.1134/S0023158418040134

[B16] Watanabe T. Oyama T. Fukumi M. (2018). Estimation of tongue motion and vowels of silent speech based on EMG from suprahyoid muscles using CNN. IEEJ Trans. Electron. Inf. Syst. 138 (7), 828–837. doi: 10.1541/ieejeiss.138.828

[B17] Williams B. M. Borroni D. Liu R. Zhao Y Zhang J Lim J. (2020). An artificial intelligence-based deep learning algorithm for the diagnosis of diabetic neuropathy using corneal confocal microscopy: a development and validation study. Diabetologia 63 (2), 419–430. doi: 10.1007/s00125-019-05023-4 31720728 PMC6946763

